# CTSE inhibits anti-tumor T cell response by promoting des-γ-carboxy prothrombin releasing in hepatocellular carcinoma

**DOI:** 10.1038/s41419-025-07753-3

**Published:** 2025-06-04

**Authors:** Yejian Wan, Xiaoxia Geng, Qianshi Liu, Shaolong Lu, Yiqiang Liu, Tao Wang, Xingmei Zhang, Na Li, Dongyun Li, Zhaoshen Li, Junjie Liu, Hong Wu, Jie Chen

**Affiliations:** 1 Department of Hepatobiliary Surgery, Affiliated Cancer Hospital of Guangxi Medical University, Nanning, China; 2 Department of Oncology & Cancer Institute, Sichuan Academy of Medical Sciences, Sichuan Provincial People’s Hospital, University of Electronic Science and Technology of China, Chengdu, Sichuan China; 3 Sichuan Cancer Hospital & Institute, Sichuan Cancer Center, School of Medicine, University of Electronic Science and Technology of China, Chengdu, Sichuan China; 4 Jinfeng Laboratory, Chongqing, China; 5 Kindstar Global Precision Medicine Institute, Wuhan, China

**Keywords:** Liver cancer, Immune evasion

## Abstract

The interactions between cancer cells and immune cells are crucial regulatory factors in forming the immuno-suppressive microenvironment. However, the underlying regulatory mechanisms remain elusive. In this study, we analyzed hepatocellular carcinoma (HCC) single-cell sequencing of public databases to investigate cellular interactions, revealing that cathepsin E (CTSE) highly expressed cancer cells exhibited significant interactions with T cells. Moreover, lower expression of CTSE is associated with an increased intra-tumoral CD3^+^ T cell infiltration. Mechanistically, CTSE highly expressed cancer cells upregulated the ubiquinone signaling pathway, enhancing the synthesis and release of des-γ-carboxy prothrombin (DCP), which subsequently activates reactive oxygen species (ROS) production and leads to apoptosis of Jurkat T cells. In vivo, animal experiments show that CTSE knockdown inhibited peripheral blood DCP levels and tumor growth while significantly enhancing the effectiveness of anti-PD-1 immunotherapy. Overall, our data reveal a regulatory mechanism involving CTSE-mediated DCP release and underscore the potential of CTSE knockdown in enhancing anti-PD-1 treatment.

**Figure:**
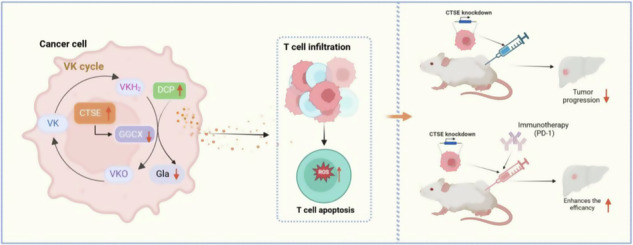
Cancer cells with high expression of CTSE upregulate the ubiquinone signaling pathway, promoting the synthesis and release of Des-γ-carboxyprothrombin (DCP). DCP can not only inhibit CD45^+^ and CD3^+^ immune infiltration, but also promote T cells to increase the production of reactive oxygen species (ROS), which leads to the increase of T cell apoptosis. CTSE knockdown can inhibit DCP levels and tumor growth, while significantly enhancing the effectiveness of anti-PD-1 immunotherapy.

Cancer cells with high expression of CTSE upregulate the ubiquinone signaling pathway, promoting the synthesis and release of Des-γ-carboxyprothrombin (DCP). DCP can not only inhibit CD45^+^ and CD3^+^ immune infiltration, but also promote T cells to increase the production of reactive oxygen species (ROS), which leads to the increase of T cell apoptosis. CTSE knockdown can inhibit DCP levels and tumor growth, while significantly enhancing the effectiveness of anti-PD-1 immunotherapy.

## Introduction

Liver cancer is one of the most common cancers and the third leading cause of cancer-related death worldwide, with hepatocellular carcinoma (HCC) accounting for 90% of cases [1–3]. The 5-year survival rate ranges from 20 to 40%, with a case fatality rate of ~70%. This is primarily attributed to late-stage diagnosis and a poor response to therapies such as chemotherapy, radiotherapy, targeted drug therapy, and immunotherapy [4, 5].

Immunotherapy has emerged as a new cornerstone in the treatment of HCC patients, but only about 30% of patients respond effectively to this therapeutic approach. The inefficacy of immune checkpoint inhibitors is linked to the “cold” tumor phenotype characterized by a lack of T cell presence in the immune-microenvironment [6, 7]. Therefore, distinguishing HCC patients with a “cold” microenvironment and transitioning them to a “hot” state is pivotal in devising effective treatment strategies.

Cathepsin E (CTSE) is an intracellular aspartic protease with hydrolytic properties in immune and gastrointestinal cells, lymphoid tissues, erythrocytes, and cancer cells [8]. The high expression of CTSE in gastrointestinal tumors, including pancreatic and HCC, serves as a critical poor prognostic marker [8, 9]. However, the functional role of CTSE appears to vary under different conditions, highlighting that CTSE can impede tumor growth and metastasis through modulation of IL-12 and endostatin, thereby influencing angiogenesis [10, 11]. Furthermore, it facilitates the surface release of tumor necrosis factor-related apoptosis-inducing ligand (TRAIL) from tumor cells, leading to tumor growth inhibition and increased apoptosis [12]. Previous studies indicate that CTSE participates in B cell lymphocyte antigen processing via the major histocompatibility complex class II (MHC-II) pathway [13]. It also plays a crucial role in B cell lymphocytes processing ovalbumin (OVA), enabling the presentation of the antigen to OVA-specific T cells [14]. The precise functions of CTSE have yet to be comprehensively elucidated. The relationship between abnormal CTSE expression in tumors and the patient’s immune microenvironment, alongside its impact on immunotherapy, remains unclear. Exploring this potential regulatory mechanism is crucial for reshaping the immune microenvironment of HCC patients and modulating the efficacy of their immunotherapy.

The currently available immune checkpoint inhibitors commonly target the adaptive immune system to induce a T-cell-mediated anti-tumor response [15, 16]. Des-γ-carboxy prothrombin (DCP), known as antagonist-II (PIVKA-II), is a vital serum biomarker for the clinical screening of HCC, emanating from HCC cells in conditions of vitamin K insufficiency or defects in gamma-carboxylase enzyme [17, 18]. Studies have demonstrated that DCP is associated with the expression of critical molecules involved in cell differentiation and immune regulation, such as sphingomyelin phosphodiesterase acid-like 3A (SMPDL3A) and glypican-3 (GPC3) [19, 20]. In clinical practice, the study demonstrated that DCP reduction >50% from baseline significantly correlates with a more favorable response to anti-PD-1 treatment, thereby improving the survival of HCC patients [21].

In this study, we applied single-cell RNA sequencing technology to clarify the cell-specific expression of *CTSE* in HCC tissue. Our findings revealed that *CTSE* was specifically highly expressed in HCC cells. We analyzed a cohort of HCC patients with differential *CTSE* expression to map the transcriptomic landscape of tumor tissues, including the tumor core and tumor immune microenvironment, by a comprehensive digital spatial profiling (DSP) approach. HCC patients with high CTSE expression activated signaling pathways for immune cell recruitment while exhibiting a systemic loss of intra-tumoral CD45^+^ immune cells. This phenomenon may be attributed to the abnormal oxidative-reduction metabolism of vitamin K and upregulated DCP expression, which could promote the apoptosis of T cells and diminish the efficacy of anti-PD-1.

## Materials and methods

### Patient sample collection

This study was approved by the Research Ethics Committee of Affiliated Cancer Hospital of Guangxi Medical University (No. KY2022003). Written informed consent was obtained from each patient. The HCC tissues of 20 patients were used for WB, or IHC, or IF, or DSP, and the basic information is provided in Supplementary Table 1. The basic information of 136 HCC patients with DCP high and low expression in this study is provided in Supplementary Table 2.

### Cell lines and cell culture

The Jurkat T cells, HepG2, Huh7, and H22 were purchased from the Chinese Academy of Sciences Cell Bank (CASCB, Beijing, China). Cells were cultured in Dulbecco’s modified Eagle’s medium (DMEM) medium (Cat. No. C11995500BT, Gibco Life Technologies, USA) or RPMI 1640 medium (Cat. No. C11875500BT, Gibco Life Technologies, USA) containing 10% FBS and 1% penicillin/streptomycin (Cat. No. 10378016, Thermo Fisher Scientific, USA) and incubated at 37 °C in a humidified atmosphere with 5% CO_2_. For Jurkat T cell activation, add 25 μL human CD3/CD8 T cell activator (Cat. No. 10911, Stemcell technologies, USA) per 1 × 10^6^/ mL were cultured for 3 days at 37 °C in a humidified atmosphere with 5% CO_2_. All cell lines tested negative for Mycoplasma bacteria as assessed by a Mycoplasma PCR Detection Kit (Cat. No. C0301S, Beyotime, China).

### Plasmids

Full-length CTSE in the CMV-MCS-3FLAG-hEF1/HTLVp-fire luciferase-T2A-puromycin vector was purchased from GeneChem (Shanghai, China) for the overexpression assay. shRNA specific for targeting CTSE was obtained from GeneChem (Shanghai, China): CTSE RNAi-1 (5′-CGTGGGAATAACCGTGTGGGA-3′), CTSE RNAi-2 (5′-GTGTGCCAACCTTAACGTCAT-3′). Human HEK-293T cells were cultured in 6-well plates until ~85% confluent and co-transfected with 2 μg of target plasmids, 1 μg of pMD2.G, and 1 μg of psPAX2 lentivirus packaging vectors using Lipofectamine 2000 (Invitrogen) according to the manufacturer’s protocol.

### Mice experiments

The 6-week-old male C57BL/6 mice were purchased from GemPharmatech (Nanjing, China). 1 × 10^6^ H22 cells with or without CTSE knockdown were orthotopically injected into the mice’s liver using microliter syringes.

For the subcutaneous tumor model, 1 × 10^6^ H22 cells with or without CTSE knockdown were injected into the C57BL/6 mice. Anti-PD-1 treatment was performed every 3 days for three times at 200 μg per mouse. Tumor volume was measured every 3 days. Half of the xenografts were cut up for flow analysis. The remaining parts were soaked in 4% paraformaldehyde for subsequent immunohistochemical staining.

### Immunohistochemistry staining

Immunohistochemistry (IHC) staining was performed on 4 μm paraffin-embedded tissue sections, which were incubated at 60 °C for 1 h and deparaffinized and rehydrated with xylene and graded alcohol, and the antigenic epitope was retrieved using Citrate-EDTA Antigen Retrieval Solution at high temperature and pressure for 4 min and cooled down to room temperature. Tissue sections were blocked with endogenous peroxidase blocking buffer for 15 min at 37 °C, followed by incubation with the primary antibodies CD8 (1:200, Cat. No. A11856, Abclonal, China), IFN-γ (1:50, Cat. No. A12450, Abclonal, China), GZMB (1:200, Cat. No. E5V2L, Cell Signaling Technology, USA), at 4 °C, overnight. After washing with PBS three times, the sections were incubated with secondary antibody for 30 min in a humid chamber at 37 °C. The sections were washed with PBS three times and performed with a DAB kit for staining. The nucleus was stained with hematoxylin. All slides were scanned using Carl Zeiss Axioscan7 microscope slide scanner at ×20 magnification. Image-Pro Plus was used to analyze the optical density of the images for quantification. The average optical density, namely, integrated optical density/ area, was calculated, and the antibody information was provided in Supplementary Table 3.

### Multiplex immunofluorescence

Multiplex immunofluorescence staining for anti-CD3e (1:100, Cat. No. Ab16669, Abcam, USA), anti-CD68 (1:100, Cat. No. 76437, Cell Signaling Technology, USA), CTSE (1:100, Cat. No. A2678, Abclonal, China) was performed using a PANO 4-plex IHC Kit (Cat. No. 10001100050, Panovue, China). Heat the repair solution in a microwave oven at high heat for 5 min, then repair the slice on low heat for 15 min, and cool down to room temperature. Tissue sections were blocked with endogenous peroxidase blocking buffer for 15 min at 37 °C. Primary antibodies were sequentially applied, followed by incubation with horseradish peroxidase-conjugated secondary antibodies. Tyramide signal amplification allowed the acquisition of multiple immunofluorescent markers. Cell nuclei were stained with 4,6-diamidino-2-phenylindole (DAPI), followed by labeling with all antigens. Fluorescence spectra were captured from 520, 570, and 650 nm under identical exposure periods. The slides were imaged using the Vectra 3.0 spectral imaging system (PerkinElmer) according to previously published instructions. The information on antibodies is provided in Supplementary Table 3.

### Western blotting

Cells were lysed in RIPA-containing protease inhibitor phenylmethane sulfonyl fluoride (PMSF). Protein was denatured at 95 °C for 10 min, electrophoretically separated on SDS–PAGE, and then transferred onto a polyvinylidene fluoride membrane. The membrane was blocked in 5% skim milk for 60 min at room temperature and incubated overnight with CTSE (1:1000, Cat. No. A2678, Abclonal, China), GGCX (1:1000, Cat. No. A1806, Abclonal, China), NOX2(1:1000, Cat. No. A19701, Abclonal, China), GAPDH (1:5000, Cat. No. 55174, Cell Signaling Technology), β-Actin (1:3000, Cat. No. AC038, Abclonal, China) at 4 °C. After being washed three times, the membrane was incubated with a secondary antibody labeled for 1 h at room temperature, washed three times. The bands were visualized using an ECL and captured using an E-Gel Imager.

### Apoptosis assay

For apoptosis assay, the suitably treated cells were washed twice with cold PBS, resuspended in binding buffer at the density of 1 × 10^6^ cells/mL, and distributed into 100 μL aliquots (1 × 10^5^) in 5 mL tubes. Five microliters of Annexin V-FITC (FXP018-100, 4A Biotech, China) was added to each tube. Cells were incubated at room temperature for 5 min in a dark environment, then added 10 μL of PI with 400 μL PBS and analyzed on a FACS Calibur (BD Biosciences, USA) immediately. Results were calculated using FlowJo software (BD Biosciences, USA).

### Flow cytometry for ROS assay

Indicated treatment was applied to Jurkat T cells and then incubated with DCFH-DA (Cat. No. C1089, Beyotime, China) or vehicle control (dimethyl sulfoxide) for 20 min at 37 °C. Invert and mix every 5 min to ensure the probe is entirely in contact with the cells. The cells were washed three times with serum-free cell culture solution to remove DCFH-DA that did not enter the cells fully. Flow cytometry was performed on a FACS Calibur (BD Biosciences, USA). The cells were then analyzed, and results were calculated using FlowJo software (BD Biosciences, USA).

### Flow cytometry for tumor tissues

Tumor tissues were chopped, digested in DF12 containing collagenase I, IV, and DNase I for 1 h at 37 °C, and filtered through a 100 μm cell strainer to generate a single-cell suspension. Cells were incubated with red blood cell lysis for 10 min at 4 °C and washed in PBS twice. Cell surface molecule staining was performed at 4 °C for 30 min in PBS in the dark. Flow cytometry was performed on a FACS Calibur (BD Biosciences, USA). The cells were then analyzed, and results were calculated using FlowJo software (BD Biosciences, USA).

### Enzyme-linked immunosorbent assay (ELISA)

Mouse serum, cell protein, and cell culture medium samples for metabolite via DCP Elisa assay analysis according to the manufacturer’s protocols.

### GeoMx^®^ digital spatial profiling (DSP) of the whole-transcriptome atlas (WTA)

Tissue microarray slides were processed following the GeoMx® DSP slide preparation user manual (MAN-10087-04). PanCK (Nanostring, GMX-RNA-MORPH-HST-12), CD45 (Nanostring, GMX-RNA-MORPH-HST-12), CD68 (Cat. No. sc-20060 AF647. Santa), and Syto83 (Nanostring, GMX-RNA-MORPH-HST-12) were used in this study. For each tissue, two pathologists selected one to three geometrical regions of interest (ROIs) with distinct cell types. Geometrical shapes, including rectangular shapes and polygons, were used to select ROIs. ROIs were placed on 20× fluorescent images scanned by GeoMx® DSP. Oligos from PanCK^+^, CD45^+^, and CD68^+^ regions were collected separately by UV-cleavage. The oligos were uniquely indexed using Illumina’s i5 × i7 dual indexing system. PCR reactions were purified, and libraries were paired-end sequenced (2 × 100) on a DNBSEQ-T7RS. Fastq files were further processed by the DND system, and raw and Q3 normalized counts of all WTA targets in each ROI were obtained through GeoMx® DSP data analysis software.

### Dimension reduction and clustering analysis

We scaled data with the top 2000 most variable genes using the FindVariableFeatures function in the R package Seurat v3. Clustering. We used variable genes for principal component analysis (PCA), used FindNeighbors in Seurat to get nearest neighbors for graph clustering based on PCs, and used FindCluster in Seurat to obtain cell subtypes, and visualized cells with the uniform manifold approximation and projection (UMAP) algorithm. To eliminate the batch effect, we performed a harmony algorithm in the Harmony R package to remove batch correction before clustering analysis.

### Quantitative real-time PCR (qRT-PCR)

For qRT-PCR quantification, briefly, 10 μL reaction mix containing TB Green® Premix Ex Taq ™ II (2×) (Cat. No. RR820Q, Takara), 400 nM of forward primer, 400 nM of reverse primer, and 1 μL sample DNA was loaded on the CFX96 Real-Time PCR Detection System (Bio-Rad). The qRT-PCR reaction was programmed as follows: denaturation at 95 °C for 30 s, followed by 40 cycles of 95 °C for 10 s and 60 °C for 30 s, and completed with a dissociation curve. All samples conducted at least three biological replicates. The primers are provided in Supplementary Table 4.

### Statistical analyses

Statistical analyses were performed using GraphPad Prism (v.9.0) (for experimental data), R (v.4.2.1), and RStudio (v.3.5.3) (for sequencing data and matched clinical variables). Comparisons between groups were conducted using *χ*^2^ tests for categorical variables. The students’ *t*-test was used to measure continuous variables. Unless otherwise noted, each experiment was repeated three or more times with biologically independent samples.

## Results

### CTSE is highly expressed in advanced HCC and is closely associated with poor prognosis of patients

**Fig. 1:**
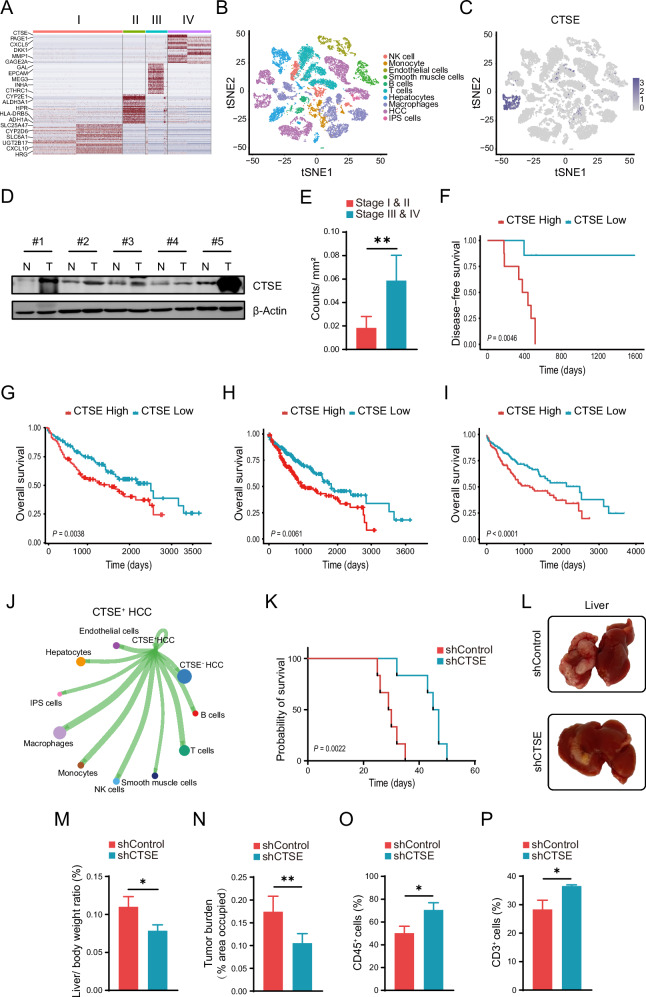
CTSE was positively correlated with HCC progression, with shorter survival and worse prognosis at high CTSE expression. **A** Heatmap of gene expression profiles across different stages of HCC. **B** Cell grouping of HCC samples based on single-cell sequencing data. **C** The expression of CTSE in different cell types in HCC tissues. **D** The western blot results reveal the expression levels of CTSE in HCC tissues and adjacent non-cancerous tissues (*n* = 5). **E** The expression level of CTSE in human HCC was analyzed by tissue microarray immunohistochemistry (*n* = 15). **F** Kaplan–Meier curve of Disease-free survival in patients with high expression and low expression of CTSE (*n* = 15). **G**–**I** Kaplan–Meier plots of overall survival of HCC patients in the high expression CTSE mRNA group and the low expression CTSE mRNA group were obtained through the Kaplan-Meier plotter and gene expression profile analysis (GEPIA) database. **J** Cell chat analysis of cell interaction. **K** Kaplan–Meier survival curves of HCC model mice injected with *CTSE* knockdown HCC cell line and control mice (*n* = 5/ group). **L** The effects of CTSE knockdown on liver morphology. **M** In situ liver model with *CTSE* knockdown group and control group were photographed with HCC in liver weight ratio (*n* = 5/ group). **N** Histogram of statistical analysis of tumor burden in orthotopic hepatocellular carcinoma model mice injected with *CTSE* knockdown group and control mice (*n* = 5/ group). Flow analysis of CD45^+^ T cells (**O**) and CD3^+^ T cells (**P**) in orthotopic hepatocellular carcinoma model mice injected with *CTSE* knockdown group and control mice. Date are resented are mean ± SEM. The *p* values are calculated by student’s t-test or one-way ANOVA. *p < 0.05; **p < 0.01; ***p < 0.001.

After utilizing single-cell transcriptomic sequencing data to compare differentially expressed genes of HCC patients with stage I, II, III, and IV, we identified the *CTSE*, which was significantly upregulated in stage IV patients of HCC (Fig. 1A). Moreover, the single-cell RNA sequencing data of 10 tumor samples were classified to 10 cell types based on specific markers, and distribution of *CTSE* positive cell sub-population is predominantly in HCC cells (Fig. 1B, C). The expression of *CTSE* in cancer cells was significantly higher in stage III and IV patients than that in stage I and II HCC patients (Supplementary Fig. S1A). We conducted western blot detecting CTSE expression in cancer tissues and adjacent normal tissues from 5 patients with HCC, and the results confirmed that CTSE expression in cancer tissues was significantly higher than that in adjacent normal tissues (Fig. 1D). IHC staining of CTSE with tissue microarray further confirmed that CTSE expression was lower in stage I and II patients compared to stage III and IV HCC patients (Fig. 1E, Supplementary Fig. S1B). Kaplan–Meier plotter analysis demonstrated that patients in the lower expression group of CTSE had longer disease-free survival compared with patients in the higher expression group (Fig. 1F). Subsequently, we demonstrated that lower CTSE expression group showed significantly longer overall survival (OS) time compared to the higher expression group using the public databases (Fig. 1G–I).

To investigate the impact of differential CTSE expression on immune cell infiltration and its regulatory role, we compared the immune cell infiltration in tumor tissues between patients with high and low CTSE expression. The results showed a significant reduction in T-cell infiltration in the tumor tissues of patients with high CTSE expression. In addition, the cell chat analysis uncovered that CTSE highly expressed cancer cells contact closely with T cells and macrophages (Fig. 1J, Supplementary Fig. S1C, D). To investigate the role of CTSE in tumor growth and its regulatory impact on the immune microenvironment, we injected CTSE knockdown H22 cells into the livers of mice, establishing an in situ liver cancer model. Kaplan-Meier survival curve showed that mice injected with CTSE knockdown have prolonged survival time (Fig. 1K). The results indicated that CTSE knockdown inhibits tumor growth, the liver/ body weight ratio, and the tumor burden of mice (Fig. 1L–N). The results of tissue flow analysis showed that compared with the control group, the ratio of CD45^+^ T cells and CD3^+^ T cells was significantly increased in the CTSE knockdown group (Fig. 1O, P). Overall, low CTSE expression in tumor cells predicts better prognosis and inhibits tumor growth by recruiting the infiltration of T cells.

### CTSE highly expressed cancer cells promote the apoptosis of T cells

**Fig. 2:**
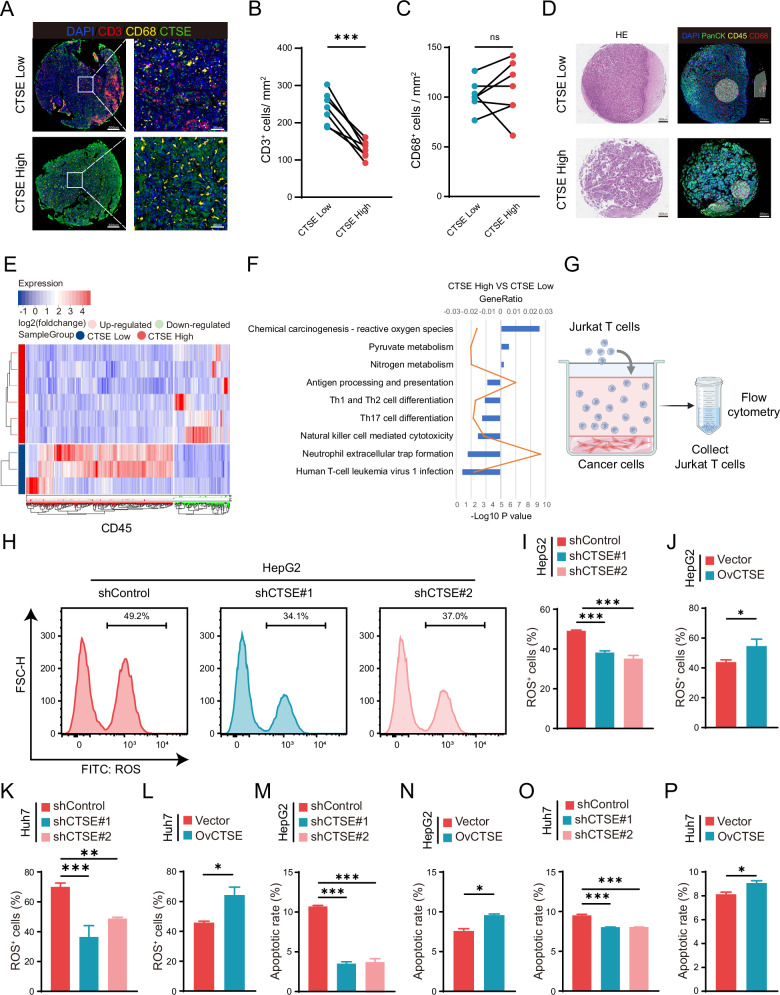
High expression of CTSE inhibits the infiltration of Jurkat T cells and induces their apoptosis through up-regulation of ROS. **A** Representative image of HCC tissue microarray staining. CD3^+^ (red), CD68^+^ (yellow), CTSE (green), DAPI (blue), (scale bar = 50 μm). Statistical analysis of the proportion of CD3^+^ T cells (**B**) and CD68^+^ macrophages (**C**). **D** Using GeoMx DSP technology analysis the regions of interest (ROIs) in the tissue sections. PanCK^+^ (green), CD45^+^ (yellow), and CD68^+^ (red) segments (scale bar = 100 μm). **E** Heatmap of differential gene expression between *CTSE* high and low expression groups in the CD45^+^ ROIs. **F** Representative Kyoto Encyclopedia of Genes and Genomes (KEGG). **G** Workflow of Jurkat T cell and cancer cell co-culture experiment. **H** Flow cytometry analysis of ROS expression in HepG2 cells after CTSE knockdown. **I**, **J** Effect of CTSE knockdown and overexpression of HepG2 cells on ROS levels of Jurkat T cells. **K**, **L** Effect of CTSE knockdown and overexpression of Huh7 cells on ROS levels of Jurkat T cells. **M**, **N** Effect of CTSE knockdown and overexpression of HepG2 cells on apoptosis levels of Jurkat T cells. **O**, **P** Effect of CTSE knockdown and overexpression of Huh7 cells on apoptosis levels of Jurkat T cells. Date are resented are mean ± SEM. The *p-*values are calculated by student’s *t*-test or one-way ANOVA. *p < 0.05; **p < 0.01; ***p < 0.001; ns not significant.

We further performed CD3, CD68, and CTSE staining on HCC tissue microarrays. Compared with patients with low expression of CTSE, the proportion of CD3^+^ T cells was significantly downregulated in the high expression of CTSE, while the proportion of CD68^+^ macrophages did not significantly change (Fig. 2A–C). We collected tissue samples from 11 HCC patients and used PanCK to mark tumor cells, CD45 to delineate the intra-tumoral immune cells, and CD68 to identify macrophages. Using GeoMx DSP technology, we selected ROIs for each sample, including tumor cells (PanCK^+^), immune cells (CD45^+^), and macrophages (CD68^+^) (Fig. 2D, Supplementary Fig. S2A). To investigate whether differential CTSE expression regulates the function and activity of CD45^+^ immune cells, we performed laser microdissection on CD45^+^ immune cells from tumor tissue samples of HCC patients and conducted RNA sequencing on ROIs. After categorizing patients based on high and low CTSE expression, Unsupervised hierarchical clustering analysis indicated that the ROIs (CD45^+^) could accurately distinguish between patients with high and low CTSE expression (Fig. 2E). KEGG pathway analysis showed that ROS production was significantly higher in the CD45^+^ immune cells from patients with high CTSE expression compared to those with low CTSE expression (Fig. 2F). Subsequently, we co-cultured HepG2 or Huh7 cell lines with either CTSE knockdown or overexpression Jurkat T cells (Fig. 2G). The results indicated that co-cultured with CTSE knockdown HepG2 or Huh7 cell lines reduced the apoptosis of cancer cells and ROS production compared to the control group. Conversely, co-cultured with CTSE overexpression HepG2 or Huh7 cells increased ROS production and the apoptosis ratio of Jurkat T cells (Fig. 2H–P, Supplementary Fig. S2B–D). Overall, CTSE highly expressed cancer cells promote the apoptosis of Jurkat T cells.

### CTSE affects DCP secreting through the vitamin K/ GGCX pathway

**Fig. 3:**
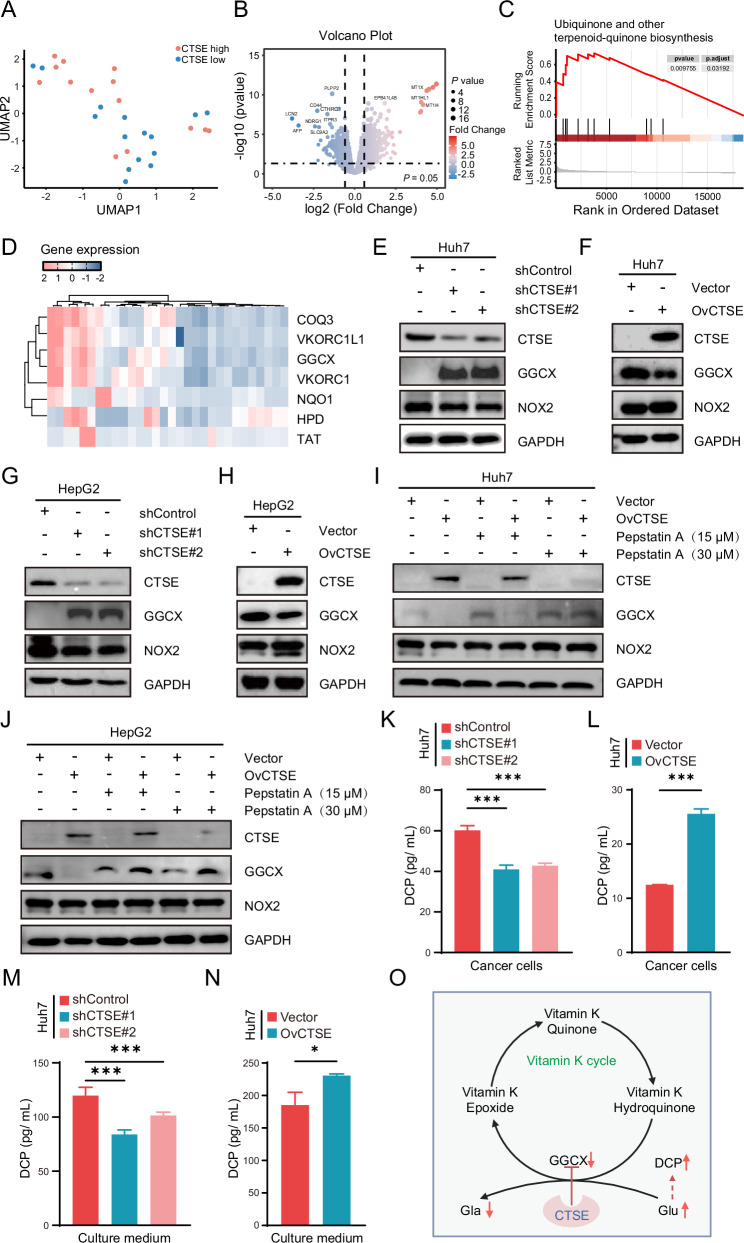
CTSE affects DCP levels by regulating the production of vitamin K cycle. **A** UMAP analysis reveals heterogeneity in PanCK^+^ cells associated with CTSE expression levels. **B** Volcano plot Showing differentially expressed genes between CTSE high and low expression patients based on PanCK analysis. **C** GSEA plots of ubiquinone signaling pathway in patients with CTSE high and low expression patients based on PanCK analysis. **D** Ubiquinone signaling pathway associated genes. Western blot was used to detect GGCX and NOX2 protein expression in Huh7 (**E**, **F**) and HepG2 cells (**G**, **H**) after CTSE knockdown and overexpression. **I**, **J** Western blot was used to detect CTSE, GGCX and NOX2 protein expression with or without pepstatin A (15 μM or 15 μM, 24 h) treatment. **K**, **L** Histogram of statistical analysis of DCP expression levels in Huh7 cell line after CTSE knockdown and overexpression. **M**, **N** Histogram of statistical analysis of DCP expression levels in Huh7 cell line culture medium after CTSE knockdown and overexpression. **O** Schematic representation of the role of CTSE in the Vitamin K cycle and its downstream effects. Date are resented are mean ± SEM. The *p-*values are calculated by student’s *t* test or one-way ANOVA. *p < 0.05; **p < 0.01; ***p < 0.001.

To further analyze how the differential expression of CTSE in tumor cells impacts patient heterogeneity, we compared gene expression changes in PanCK^+^ cells from patients with high and low CTSE expression. UMAP showed the separation of CTSE high and CTSE low ROIs (Fig. 3A). Further analysis revealed that, compared to patients with high CTSE expression, those with low CTSE expression exhibited multiple upregulated and downregulated gene expressions (Fig. 3B). Gene Set Enrichment Analysis (GSEA) of DEGs in cancer cells revealed that the differential expression of *CTSE* was closely related to the activation of the ubiquinone signaling pathway (Fig. 3C, D). In HepG2 and Huh7 cell lines with CTSE knockdown, we found that the expression levels of γ-glutamyl carboxylase (GGCX) were significantly elevated, while the expression level of NADPH oxidase 2 (NOX2) showed no significant changes. Conversely, overexpression of CTSE in HepG2 and Huh7 cell lines yielded the opposite results (Fig. 3E–H). Treatment of CTSE overexpression cells with pepstatin A suppressed CTSE expression and induced a marked upregulation of GGCX (Fig. 3I, J). We further assessed the levels of DCP following CTSE knockdown and overexpression, revealing that the intracellular and culture medium DCP levels were significantly reduced in CTSE knockdown HCC cell lines, while DCP levels markedly increased after overexpression (Fig. 3K–N). Collectively, our results demonstrated that CTSE can regulate the generation of abnormal prothrombin DCP in cancer cells by affecting the activity of the ubiquinone signaling pathway in HCC cells (Fig. 3O).

### CTSE highly expressed HCC cells release DCP to promote apoptosis of Jurkat T cell

**Fig. 4:**
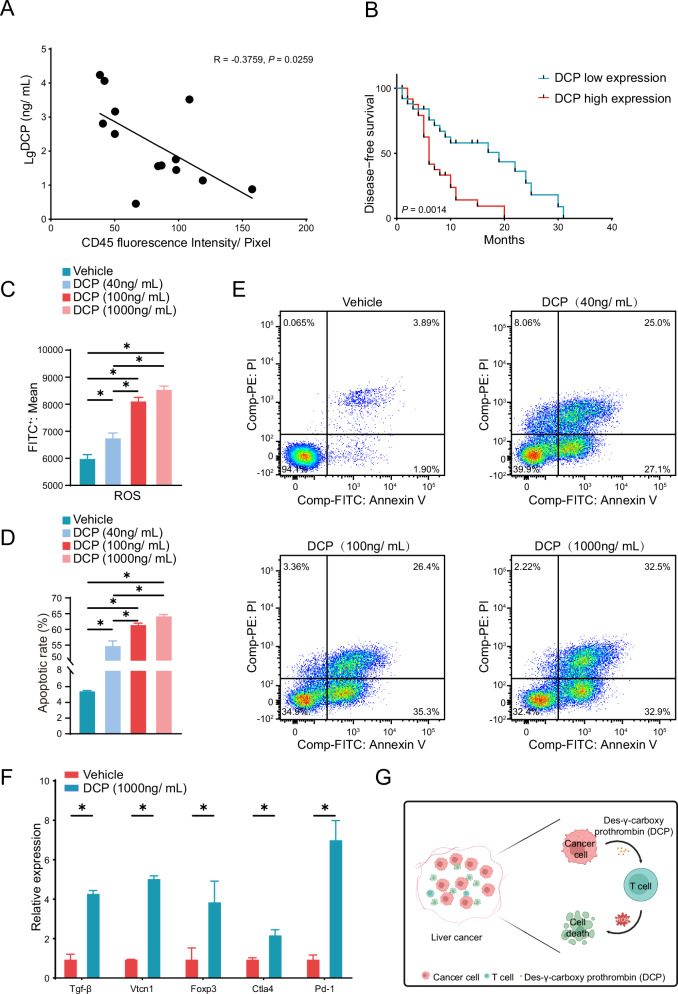
HCC cells with high expression of CTSE accelerate ROS expression and induce apoptosis of Jurkat T cells by releasing DCP. **A** Correlation between DCP levels and CD45^+^ immune cell infiltration in HCC patients. **B** Kaplan–Meier analysis of Disease-free survival in patients with DCP low expression and DCP high expression. ROS production (**C**) and apoptosis (**D**, **E**) of Jurkat T cells treated with sterile water or different concentrations of DCP (40 ng/ mL, 100 ng/ mL, 1000 ng/ mL). **F** qRT-PCR analysis of indicated genes in Jurkat T cells treated with or without DCP (1000 ng/mL). **G** Diagram of the effect of DCP released by tumor cells on Jurkat T cells. Date are resented are mean ± SEM. The *p-*values are calculated by student’s *t* test or one-way ANOVA. *p < 0.05; **p < 0.01; ***p < 0.001.

We conducted a correlation analysis of DCP levels in the peripheral blood of 32 patients with HCC and the infiltration of CD45^+^ immune cells in tumor tissues, revealing a significant negative correlation between DCP levels and immune cell counts (Fig. 4A). Prognostic data from 120 HCC patients indicated that DCP levels could serve as a predictive marker for the prognosis of HCC patients, with DCP high expression correlating with poorer outcomes (Fig. 4B). We speculated whether elevated CTSE expression contributes to the secretion of DCP, thereby promoting the apoptosis of Jurkat T cells in the microenvironment. We treated Jurkat T cells with different concentrations of DCP, and the results showed that DCP significantly increased ROS levels and apoptosis of Jurkat T cells (Fig. 4C–E). We further examined the expression of T cell exhaustion biomarkers using qPCR, identifying that DCP treatment induced PD-1, CTLA4, FOXP3, VTCN-1, and TGF-β expression (Fig. 4F). In summary, DCP plays a role in regulating the biological process of Jurkat T cells apoptosis by modulating ROS levels (Fig. 4G).

### Inhibiting CTSE expression in cancer cells enhances the efficacy of anti-PD-1 for HCC

**Fig. 5:**
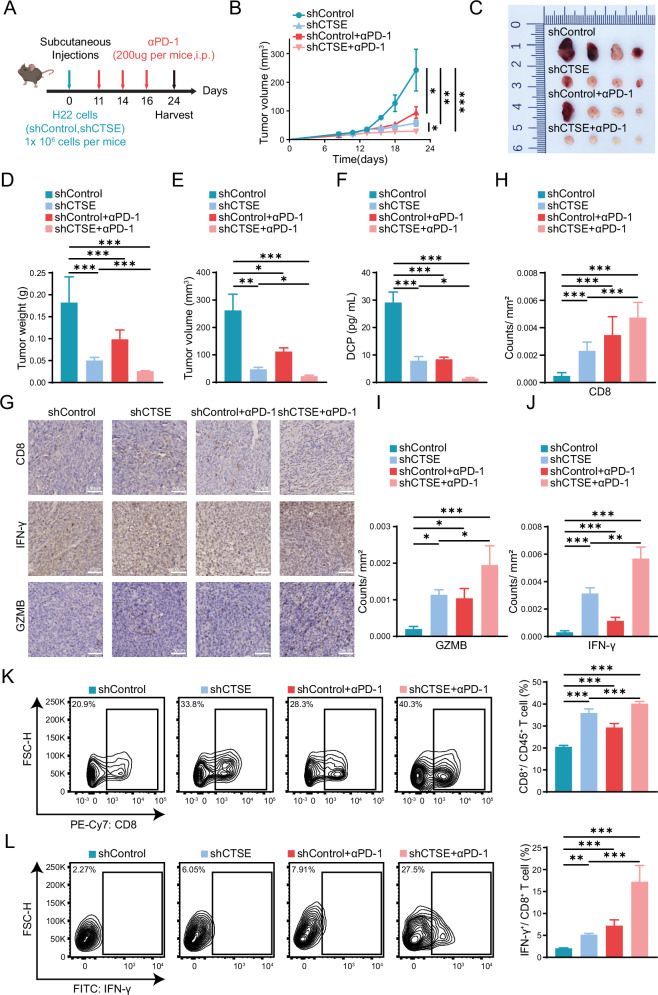
CTSE knockdown reduce DCP levels and enhance the immunotherapy of liver cancer in mice. **A** Experimental design for subcutaneous tumor model using CTSE knockdown H22 cells and anti-PD-1 treatment (*n* = 5/ group). **B** Tumor volume growth curve in different groups with CTSE knockdown and anti-PD-1 immunotherapy (*n* = 5/ group). Tumor image (**C**), tumor weight (**D**), and tumor volume (**E**) in different groups with CTSE knockdown and anti-PD-1 immunotherapy (*n* = 5/ group). **F** DCP concentration in different groups mice blood with CTSE knockdown and anti-PD-1 immunotherapy (*n* = 3/ group). **G** Immunohistochemical (IHC) analysis of CD8, IFN-γ, and GZMB expression in tumor tissues with CTSE knockdown and anti-PD-1 immunotherapy (*n* = 5/ group). Immunofluorescence staining of CD8 (**H**), GZMB (**I**), IFN-γ (**J**) was statistically analyzed in each group. **K**, **L** Flow cytometry analysis of CD8^+^ T cells and IFN-γ^+^CD8^+^ T cells in tumor tissues. Date are resented are mean ± SEM. The *p-*values are calculated by student’s *t* test or one-way ANOVA. *p < 0.05; **p < 0.01; ***p < 0.001.

To further investigate the role of CTSE expression in intra-tumoral immune cell infiltration, we constructed a subcutaneous tumor model in mice using the CTSE knockdown H22 cells (Fig. 5A). We measured tumor size and weight, and the results demonstrated that CTSE knockdown inhibited tumor growth of liver cancer cells. Additionally, the effectiveness of anti-PD-1 immunotherapy was significantly enhanced in the CTSE knockdown group, resulting in a synergistic therapeutic effect (Fig. 5B–E). We also assessed the levels of DCP in the CTSE knockdown mice, revealing that the blood DCP levels were reduced. Moreover, the DCP levels after anti-PD-1 immunotherapy were significantly decreased (Fig. 5F). Analysis of IHC staining showed that the expression levels of CD8, IFN-γ, and GZMB were significantly upregulated in tumor tissues with CTSE knockdown and the anti-PD-1 combination treatment group (Fig. 5G–J). CTSE knockdown exhibited a significant reduction in vasculature (Supplementary Fig. S3A). Multicolor flow cytometry analysis revealed a significant intra-tumoral infiltration of CD8^+^ and IFNγ^+^CD8^+^ T cells in the tumor tissues of the CTSE knockdown group and anti-PD-1 combination treatment group (Fig. 5K, L, Supplementary Fig. S3B). Overall, HCC cells with high CTSE expression releases a higher level of DCP, which will affect anti-PD-1 immunotherapy by inhibiting the intra-tumoral infiltration of CD8^+^ and IFNγ^+^CD8^+^ T cells.

## Discussion

The heterogeneity of tumor cells and the tumor immune microenvironment, organized by various immune and stromal cells, contributes to tumor metastasis, relapse, and drug resistance [22–24]. The linkage between the distinct subtypes within the tumor immune microenvironment and the clinical relevance of HCC remains unclear. Here, we utilized single-cell RNA sequencing combined with spatial transcriptomics to reveal the role of cancer cell-expressed CTSE in HCC progression and immune microenvironment regulation. We found that HCC patients with high CTSE expression had poor prognosis. Cancer cells with high CTSE expression promote the upregulation of DCP in HCC by suppressing ubiquinone signaling activation and regulating the redox capacity of vitamin K. The release of DCP from HCC cells is a key regulatory factor in forming a T cell-depleted tumor immune microenvironment.

CTSE has been recognized as a promising prognostic biomarker for multiple cancers, such as pancreatic ductal adenocarcinoma (PDAC) [25, 26], gastric [27], esophageal [28], bladder [29, 30], cholangiocarcinoma [31], rectal cancer [32], and breast cancer [33]. Here, we report that CTSE is significantly upregulated in patients with advanced liver cancer and that patients with high CTSE expression have significantly shorter progression-free survival and overall survival, which is consistent with our findings. In conclusion, CTSE is closely associated with poor prognosis of HCC patients and can be used as an independent prognostic marker for HCC.

CTSE was found to have another function in modulating immune cells, serving as the major aspartic protease involved in antigen processing via MHC-II pathway in a B cell lymphoblast cell line [13, 14]. In this study, we found that the tumor tissues of patients with high CTSE expression tended to exhibit a “cold” tumor microenvironment characterized by the absence of CD45^+^ immune cells. Our unbiased DSP approach identified that *CTSE* highly expressed HCC cells activated the ROS signaling pathway to induce apoptosis of Jurkat T cells. Collectively, our data suggested that *CTSE* highly expressed cancer cells eliminate CD45^+^ immune cells, especially T cells, leading to a systemic immunosuppressive microenvironment. This key finding provides insights into the etiology of immune deserts in human HCC.

DCP is an abnormal prothrombin secreted by tumor cells of primary HCC. It has been identified that one or more glutamate residues in DCP undergo post-translational carboxylation to form γ-glutamic acid [17, 34, 35]. We uncovered that CTSE high expressed cancer cells secreted more DCP through the dysregulated ubiquinone signaling pathway. Moreover, DCP released by cancer cells could induce apoptosis of Jurkat T cells and upregulate the expression of exhausted biomarkers on Jurkat T cells. This may be an important reason for the immunosuppressive microenvironment in CTSE highly expressed HCC patients, leading to an immune desert state.

Considering that the infiltration of CD8^+^ T cells in the microenvironment is an important indicator for evaluating the efficacy of immunotherapy [36, 37], patients with low CTSE expression may experience better outcomes from immunotherapy. Additionally, targeting DCP to remodel the immunosuppressive microenvironment may enhance the treatment efficacy of immunotherapy and provide a novel therapeutic strategy for HCC patients.

In conclusion, this study provides a framework for understanding the spatial and functional heterogeneity of HCC in patients with differential CTSE expression. It reveals the potential molecular mechanisms by which CTSE influences the generation of DCP in cancer cells by activating the ubiquinone signaling pathway. DCP promotes apoptosis of Jurkat T cells within the microenvironment to form an immune desert state. This research offers a valuable resource for exploring the strategies to enhance the efficacy of immunotherapy, particularly in patients with high CTSE expression.

## Supplementary information

Western blot

Supplementary material

## Data Availability

All datasets are available from the corresponding author on reasonable request.
