# Supplementary material for: CTSE inhibits anti-tumor T cell response by promoting des-γ-carboxy prothrombin releasing in hepatocellular carcinoma
Source: Cell Death Dis. 2025 Jun 4;16(1):434. doi: 10.1038/s41419-025-07753-3 (PMC12137924; doi:10.1038/s41419-025-07753-3)

Fig. 1D

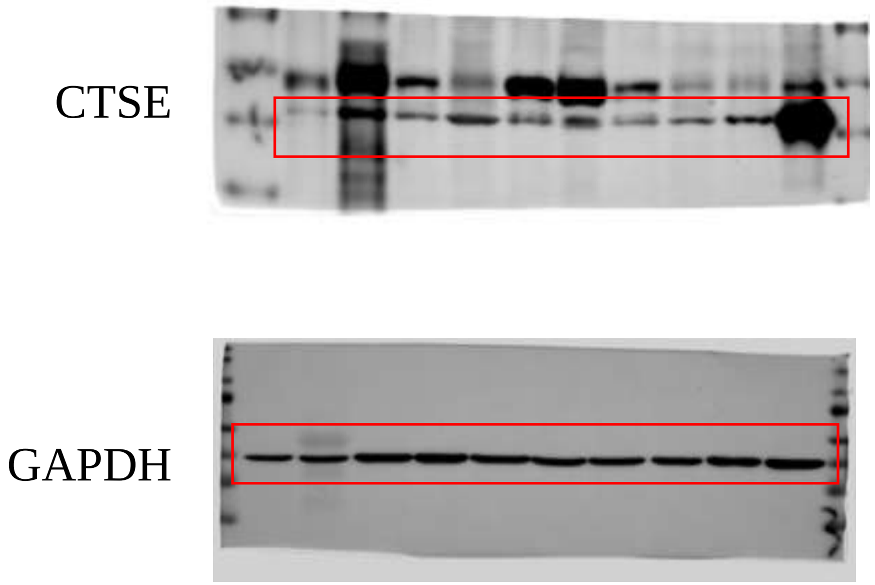

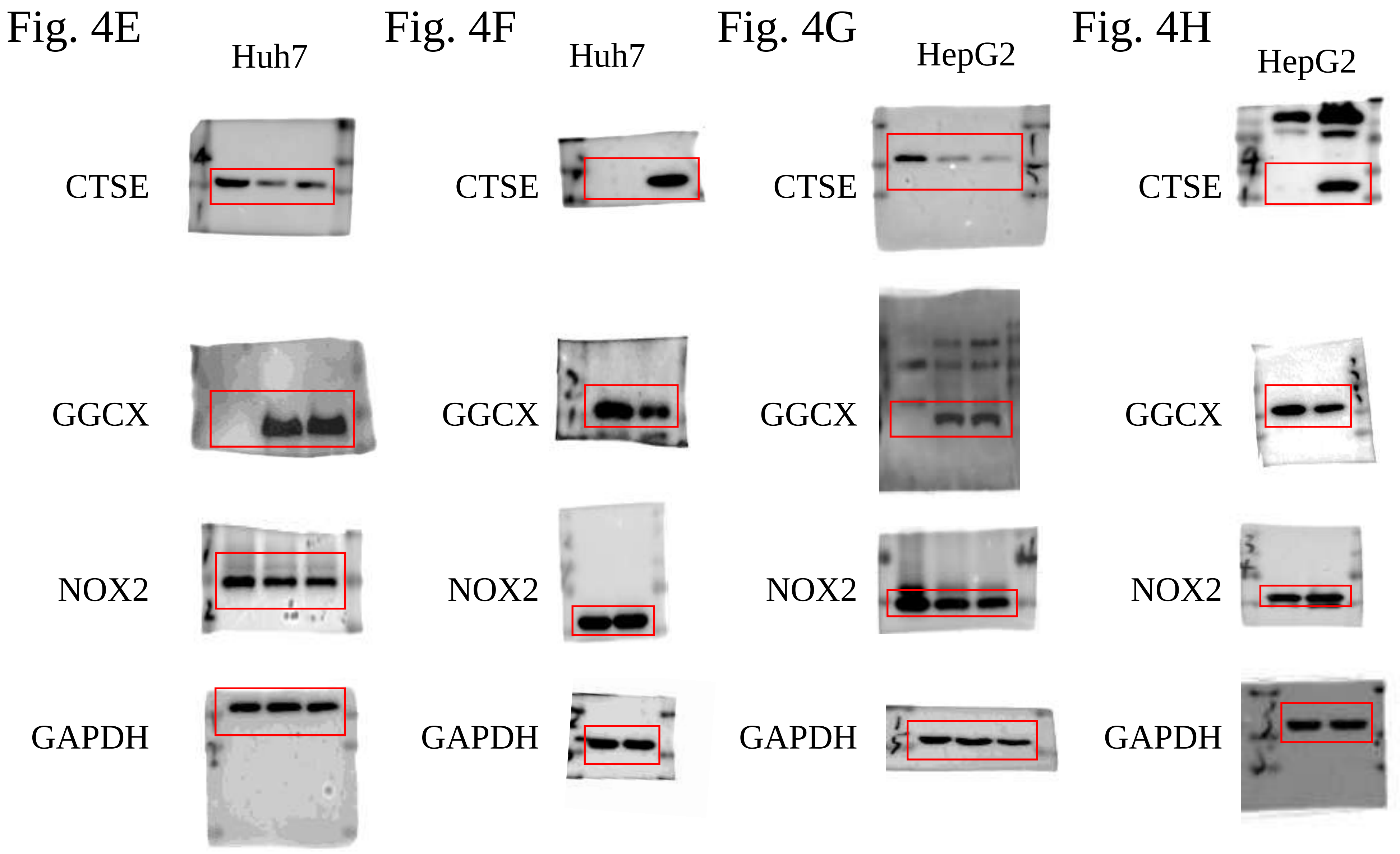
Fig. 4E
Huh7
Fig. 4F
Huh7
Fig. 4G
HepG2
Fig. 4H
HepG2
CTSE
GGCX
NOX2
GAPDH
CTSE
GGCX
NOX2
GAPDH
CTSE
GGCX
NOX2
GAPDH
CTSE
GGCX
NOX2
GAPDH

Fig. 4I

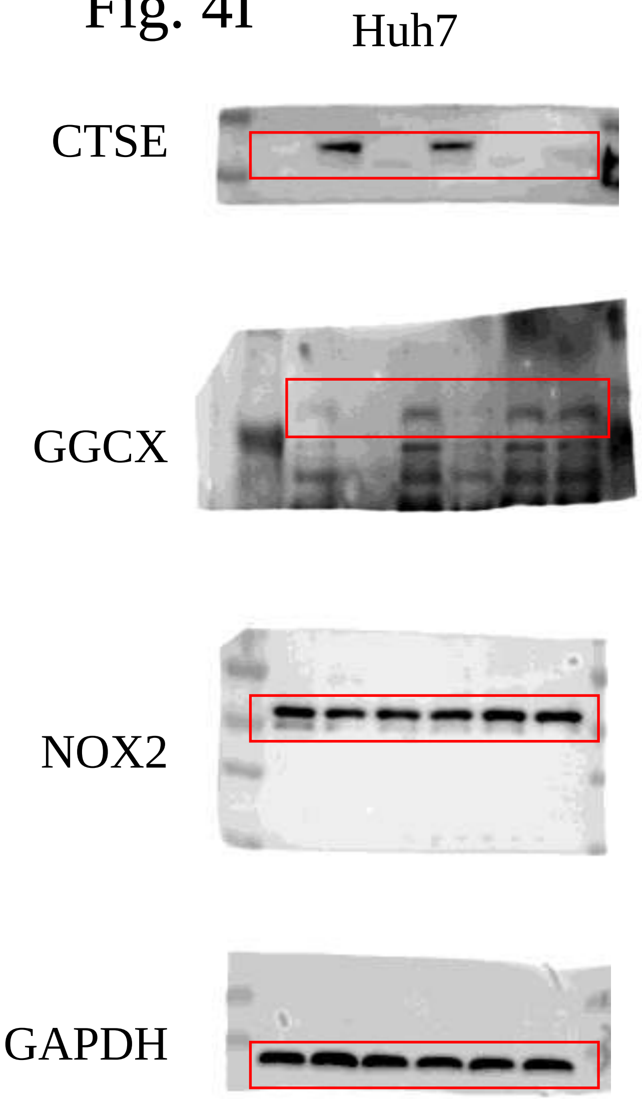

Fig. 4J

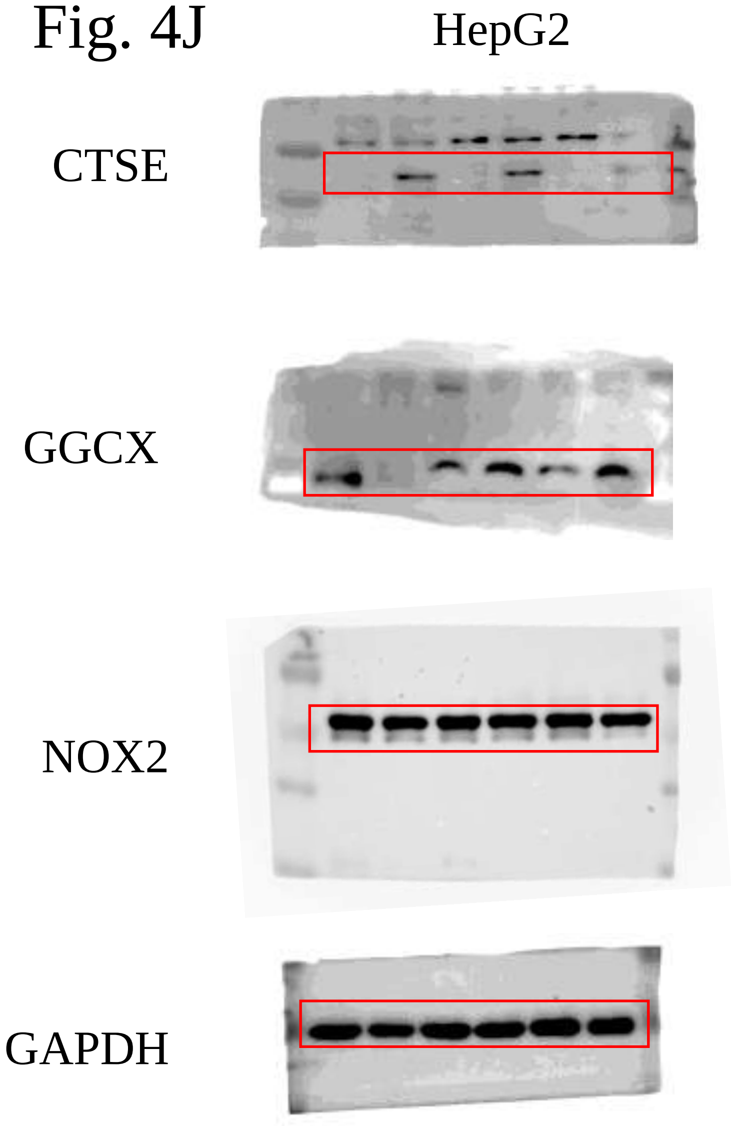

Supplement: Supplementary file 1 — Western blot [file 41419_2025_7753_MOESM1_ESM.pdf]
