# Supplementary material for: CTSE inhibits anti-tumor T cell response by promoting des-γ-carboxy prothrombin releasing in hepatocellular carcinoma
Source: Cell Death Dis. 2025 Jun 4;16(1):434. doi: 10.1038/s41419-025-07753-3 (PMC12137924; doi:10.1038/s41419-025-07753-3)
Supplement: Supplementary file 2 — Supplementary material [file 41419_2025_7753_MOESM2_ESM.pdf]

Supplementary Table 1. Clinical characteristics of hepatocellular carcinoma (HCC) patients.

Patients' No.	Age	Gender	Pathologic type	CNLC[1]	BCLC[2]	Using	AFP[3]（0.89-8.78ng/ml）
1	47	Male	HCC[4]	IIIa	B	IHC[5]/ IF[6]/ DSP[7]	2.07
2	60	Male	HCC	IIIa	B	IHC/ IF/ DSP	17.19
3	38	Male	HCC	Ib	A	IHC/ IF/ DSP	>1200
4	60	Male	HCC	Ia	A	IHC/ IF/ DSP	119
5	45	Female	HCC	IIIa	A	IHC/ IF/ DSP	>2000
6	47	Male	HCC	Ib	A	IHC/ IF/ DSP	1.61
7	56	Male	HCC	Ib	A	IHC/ IF/ DSP	375.19
8	56	Male	HCC	Ia	A	IHC/ IF/ DSP	1738.21
9	50	Male	HCC	IIa	B	IHC/ IF/ DSP	9.13
10	52	Female	HCC	Ib	A	IHC/ IF/ DSP	28.68
11	42	Male	HCC	Ia	A	IHC/ IF/ DSP	3.38
12	51	Male	HCC	Ia	A	IHC/ IF/ DSP	4.46
13	56	Male	HCC	Ia	A	IHC/ IF/ DSP	17.89
14	64	Male	HCC	Ib	A	IHC/ IF/ DSP	29.48
15	56	Male	HCC	Ia	A	IHC/ IF/ DSP	2.8
16	71	Male	HCC	Ib	A	WB[8]	11.92
17	67	Male	HCC	Ib	A	WB	1.46
18	49	Male	HCC	IIa	B	WB	12.06
19	72	Male	HCC	Ib	A	WB	2.4
20	44	Male	HCC	Ia	A	WB	2.75

1.CNLC: China National Logging Corporation. **2. BCLC:** Barcelona Clinic Liver Cancer. **3. AFP:** Alpha-fetoprotein. **4. HCC:** Hepatocellular carcinoma. **5. IHC:** Immunohistochemistry. **6. IF:** Immunofluorescence**. 7. DSP:** Digital spatial Profiling**. 8. WB:** Western blotting.

Supplementary Table 2. Clinical characteristics of hepatocellular carcinoma (HCC) patients with DCP high and low expression.

Patients' No.	Age	Gender	Pathologic type	CNLC[1]	BCLC[2]
1	52	Male	HCC[3]	Ib	A
2	59	Male	HCC	IIa	B
3	46	Female	HCC	IIIb	C
4	60	Male	HCC	IIIb	C
5	36	Male	HCC	Ib	A
6	55	Male	HCC	IIIa	C
7	45	Male	HCC	Ib	A
8	61	Male	HCC	IIIa	C
9	56	Male	HCC	Ia	A
10	43	Male	HCC	IIIa	C
11	44	Male	HCC	IIb	B
12	72	Male	HCC	IIIa	C
13	29	Male	HCC	Ib	A
14	58	Male	HCC	IIIa	C
15	47	Male	HCC	Ia	A
16	61	Male	HCC	IIIb	C
17	55	Male	HCC	IIIa	C
18	54	Male	HCC	Ib	A
19	63	Male	HCC	IIIb	C
21	55	Male	HCC	Ib	A
22	35	Male	HCC	Ia	A
23	48	Male	HCC	Ia	A
24	66	Male	HCC	Ia	A
25	76	Male	HCC	Ia	A
26	58	Male	HCC	IIIa	C
27	68	Male	HCC	Ia	A
28	59	Male	HCC	Ia	A
29	58	Male	HCC	Ib	A
30	58	Male	HCC	Ia	A
31	54	Female	HCC	Ia	A
32	58	Male	HCC	Ia	A
33	58	Male	HCC	IIIa	B

34	62	Male	HCC	Ib	A
35	39	Female	HCC	IIa	B
36	42	Male	HCC	Ia	A
37	59	Male	HCC	Ia	A
38	35	Male	HCC	Ib	A
39	39	Male	HCC	Ib	A
40	37	Male	HCC	IIa	B
41	72	Male	HCC	Ib	A
42	53	Male	HCC	Ia	A
43	47	Male	HCC	Ib	A
44	57	Male	HCC	IIIb	C
45	46	Male	HCC	IIb	B
46	67	Male	HCC	IIIb	C
47	58	Male	HCC	IIIa	C
48	35	Male	HCC	IIIa	C
49	70	Male	HCC	IIIa	C
50	57	Male	HCC	Ib	A
51	40	Male	HCC	Ib	A
52	41	Male	HCC	IIIa	C
53	67	Male	HCC	Ib	A
54	33	Male	HCC	IIa	B
55	74	Male	HCC	IIa	B
56	41	Male	HCC	IIIa	C
57	49	Male	HCC	IIb	B
58	46	Male	HCC	IIIa	C
59	59	Male	HCC	Ia	A
60	57	Male	HCC	IIIa	C
61	56	Male	HCC	IIIa	C
62	59	Female	HCC	Ia	A
63	40	Male	HCC	Ia	B
64	56	Male	HCC	IIIa	C
65	46	Male	HCC	IIIa	C
66	37	Male	HCC	IIIa	C
67	66	Male	HCC	Ia	A
68	54	Male	HCC	Ia	A
69	71	Male	HCC	IIIa	C
70	53	Male	HCC	IIIa	C
71	50	Male	HCC	Ib	A
72	67	Male	HCC	IIIa	C

73	58	Female	HCC	IIIa	C
74	60	Male	HCC	Ia	A
75	38	Male	HCC	IIb	B
76	62	Female	HCC	IIa	B
77	36	Male	HCC	IIIa	C
78	56	Male	HCC	Ia	A
79	44	Male	HCC	IIIa	C
80	49	Male	HCC	Ia	A
81	40	Male	HCC	Ia	A
82	36	Male	HCC	Ib	A
83	54	Male	HCC	Ia	A
84	54	Male	HCC	Ia	A
85	69	Male	HCC	Ib	A
86	48	Male	HCC	Ib	A
87	58	Male	HCC	Ib	A
88	40	Male	HCC	Ia	A
89	41	Male	HCC	Ia	A
90	67	Male	HCC	Ib	A
91	56	Male	HCC	IIa	B
92	59	Male	HCC	Ia	A
93	40	Male	HCC	Ia	A
94	56	Female	HCC	Ia	A
95	42	Female	HCC	Ia	A
96	37	Male	HCC	Ia	A
97	71	Female	HCC	Ib	A
98	61	Female	HCC	Ia	A
99	40	Male	HCC	Ia	A
100	39	Male	HCC	Ia	A
101	63	Female	HCC	Ia	A
102	62	Male	HCC	Ia	B
103	68	Male	HCC	IIa	B
104	51	Male	HCC	Ib	A
105	50	Male	HCC	Ia	A
106	65	Male	HCC	Ia	A
107	54	Female	HCC	Ib	A
108	48	Female	HCC	Ib	A
109	42	Male	HCC	Ib	A
110	66	Male	HCC	IIIb	C
111	70	Male	HCC	IIIa	C

112	59	Male	HCC	Ib	A
113	37	Male	HCC	Ib	A
114	63	Female	HCC	IIIb	C
115	59	Male	HCC	IIa	B
116	49	Male	HCC	Ib	A
117	78	Male	HCC	Ib	A
118	59	Female	HCC	Ia	A
119	37	Male	HCC	Ia	A
120	47	Male	HCC	Ib	A
121	55	Male	HCC	IIIa	C
122	34	Male	HCC	Ib	A
123	66	Male	HCC	Ib	A
124	44	Female	HCC	Ib	A
125	68	Male	HCC	Ia	A
126	66	Male	HCC	Ia	A
127	75	Female	HCC	Ib	A
128	59	Male	HCC	IIa	B
129	53	Male	HCC	Ia	A
130	59	Female	HCC	Ia	A
131	56	Male	HCC	Ia	A
132	50	Female	HCC	Ia	A
133	68	Male	HCC	Ib	A
134	46	Male	HCC	Ia	A
135	69	Male	HCC	Ib	A
136	54	Male	HCC	Ia	A

1. CNLC: China National Logging Corporation. **2. BCLC:** Barcelona Clinic Liver Cancer. **3. HCC:** Hepatocellular carcinoma.

Supplementary Table 3. Reagents and antibodies

No	Catalog	Antibody	Sources	Dilution	Corp.
1	A2678	CTSE	Rabbit	1:1000 (for WB)	Abclonal
2	5174	GAPDH	Rabbit	1:5000 (for WB)	Cell Signaling Technology
3	AC038	β-Actin	Rabbit	1:3000 (for WB)	Abclonal
4	A1806	GGCX	Rabbit	1:1000 (for WB)	Abclonal
5	A19701	NOX2	Rabbit	1:1000 (for WB)	Abclonal
6	A11856	CD8	Rabbit	1:200 (for IHC)	Abclonal
7	E5V2L	GZMB	Rabbit	1:200 (for IHC)	Cell Signaling Technology
8	A12450	IFN-γ	Rabbit	1:50 (for IHC)	Abclonal
9	Ab16669	CD3e	Rabbi	1:100 (for IF)	Abcam
10	76437	CD68	Rabbi	1:100 (for IF)	Cell Signaling Technology
11	13917	CD45	Rabbi	1:100 (for IF)	Cell Signaling Technology
12	Ab7753	PancK	Mouse	1:100 (for IF)	Abcam
13	550954	BD Pharmingen™ PerCP-Cy™5.5 Rat Anti-Mouse CD4	Rat	1：100	BD Biosciences
14	1031160	APC/Cyanine7 Anti-mouse CD45 antibody	Rat	1：100	Biolegend
15	746776	BD OptiBuild™ BV480 Hamster Anti-Mouse CD3e	Hamster	1：100	BD Biosciences
16	100722	PE/Cyanine7 Anti-mouse CD8a antibody	Rat	1：100	Biolegend
17	557724	BD Pharmingen™ Alexa Flour 488 Rat Anti-Mouse IFN-γ	Rat	1：100	BD Biosciences
18	C8160	Collagenase Type IV		1.5 mg/ml	Solarbio
19	C8140	Collagenase Type I		1.5 mg/ml	Solarbio
20	C0065	DAPI solution			Solarbio
21	11668019	LipofectmineTM 2000 Transfection Reagent			ThermoFisher SCIENTIFIC

Supplementary Table 4. Sequences of the primers used for qRT-PCR.

Gene	Primer sequence (5’→3’)	Amplification size (bp)
CXLC1	F: TGGCTTAGAACAAAGGGGCTT	107
	R: GGTAGCCCTTGTTTCCCCC	
CXCL5	F: TGTGCAATTAACAAAGCTACTGC	128
	R: AGGCATCTAAAAAGCTCAGCA	
CXCL8	F: CTCCAAACCTTTCCACCCCA	174
	R: TTCTCAGCCCTCTTCAAAAACT	
CCL2	F: CAGCAGCAAGTGTCCCAAAG	127
	R: CGGAGTTTGGGTTTGCTTGT	
CCL20	E: TCCTGGCIGCTTIGATGICA	69
	R: CAAAGTTGCTIGCTGCTTCTGA	
TGFβ	F: GGAAATTGAGGGCTTTCGCC	92
	R: CGGTAGTGAACCCTGCGTTG	
VTCN1	F: GCTAAAGAGCCACAGATGGGT	140
	R: TGATGGAGTGTCTCCCTGAAA	
GAPDH	F: CAAGCTCATTTCCTGGTATGAC	142
	R: CAGTGAGGGTCTCTCTCTTCCT	
FOXP3	F: GGCACAATGTCTCCTCCAGAGA	128
	R: CAGATGAAGCCTTGGTCAGTGC	
CTLA4	F: ACGGGACTCTACATCTGCAAGG	121
	R: GGAGGAAGTCAGAATCTGGGCA	
PD-1	F: CAGTTCCAAACCCTGGTGGT	114
	R: GGCTCCTATTGTCCCTCGTG	

Supplementary Fig. 1

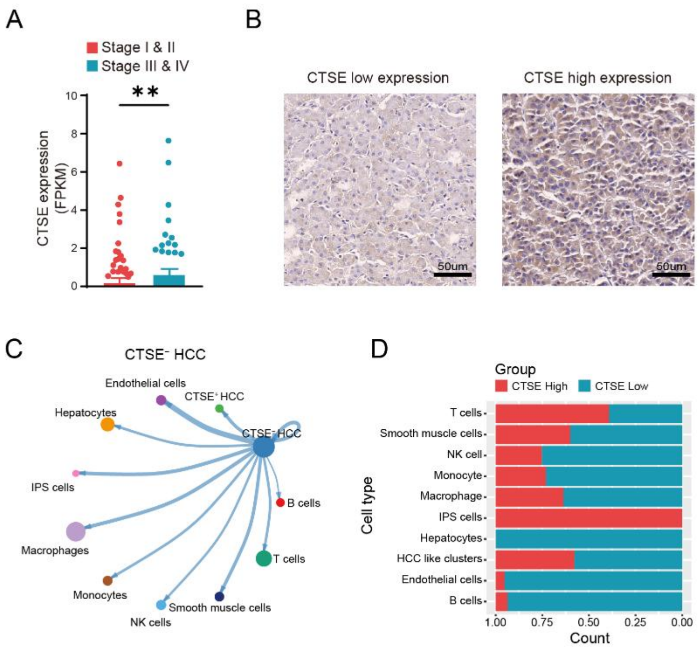

Supplementary Fig. 1 CTSE high expression in stage III and IV of HCC. A CTSE expression levels in stages I, II and stages III, IV of HCC. **B** Immunohistochemical analysis of CTSE expression in HCC tissues. **C** Cellular interactions between CTSE expressing HCC cells and other cell types in the tumor microenvironment. **D** Distribution of cell types in CTSE high and CTSE low HCC groups. Date are resented are mean ± SEM. The *p* values are calculated by student's t-test or one-way ANOVA. **p* < 0.05; ***p* < 0.01; ****p* < 0.001.

Supplementary Fig. 2

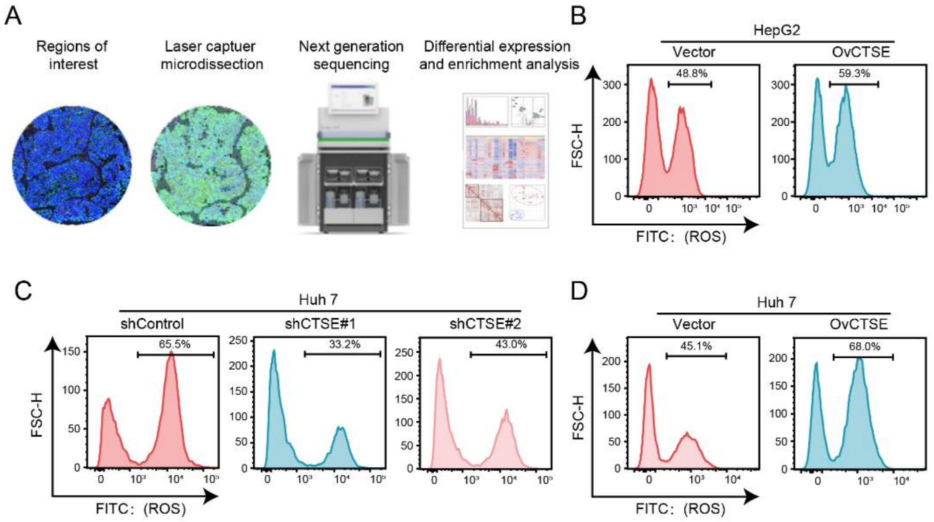

Supplementary Fig. 2 CTSE induces ROS release. A Workflow for identifying differentially expressed genes in ROIs within HCC tissues. **B** Effects of CTSE overexpression and knockdown on ROS levels in HepG2 and Huh7 cells.

Supplementary Fig. 3

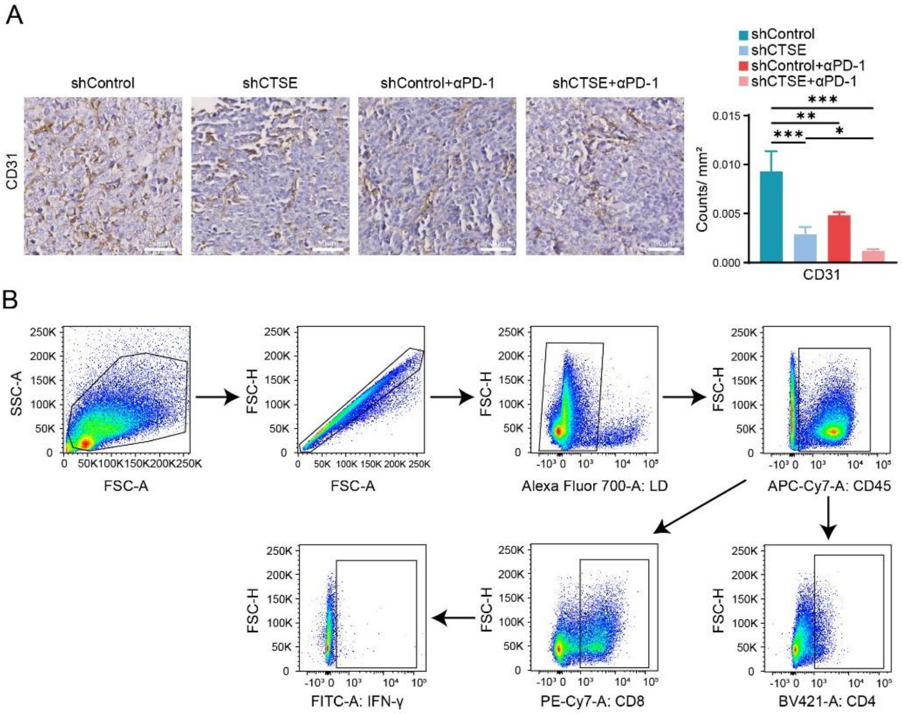

Supplementary Fig. 3 Immunohistochemical and Flow cytometry process of mice tumor tissues immune cells. A Immunohistochemical analysis of CD31 expression in tumor tissues with CTSE knockdown and anti-PD-1 immunotherapy (n=5/ group). **B** Gating strategy of flow cytometry in tumor tissues of mice.
